# AD-RISK FACTORS AND TIMED UP AND GO TASKS IN IMPAIRED AND UNIMPAIRED OLDER ADULTS

**DOI:** 10.1093/geroni/igae098.2599

**Published:** 2024-12-31

**Authors:** Ashley Price, Megan Stradtman, Jessica Alber, Maeve Durkin

**Affiliations:** University of Rhode Island, Kingston, Rhode Island, United States; Butler Hospital, Providence, Rhode Island, United States; University of Rhode Island, Kingston, Rhode Island, United States; University of Rhode Island, Kingston, Rhode Island, United States

## Abstract

The Timed Up and Go (TUG) is a clinical assessment tool evaluating mobility and dual-task function. This study examined TUG performance in a sample of cognitively unimpaired (CU) older adults at low risk (LR)/high risk (HR) for Alzheimer’s disease (AD) and a group of mild cognitive impairment (MCI) patients. 194 older adults aged 55-80 (82 M, 112 F) comprised 3 groups: 75 CU LR, 76 CU HR, and 32 MCI. Having at least one copy of the APOE E4 allele designated CU participants as high risk for AD. The TUG is a 20-foot walking task, and TUG-DT adds serial subtractions to the original task to determine dual-task cost (DTC). Both TUG and TUG-DT tasks were completed (2 runs each) during clinical assessment. DTC was calculated for both number of steps and time to completion to determine the change in performance from single to dual task. Univariate ANOVA assessed the group differences in key TUG outcome variables (number of steps, time to completion). Though the completion time for TUG-run1 was as expected (LR< HR< MCI), practice effects were suspected in TUG-run2 as group completion times were not significantly different. Additionally, significant differences were identified in TUG-DT time between LR and MCI (p=< 0.001) and HR and MCI (p=0.003). There were significant differences in TUG/TUG-DT steps between the LR and HR groups and LR and MCI. This suggests improved gait efficiency in the LR CU group, compared to both the HR and the patient group. DTC for time or steps showed no significant group differences.

